# Abnormal Elastic and Vibrational Behaviors of Magnetite at High Pressures

**DOI:** 10.1038/srep06282

**Published:** 2014-09-04

**Authors:** Jung-Fu Lin, Junjie Wu, Jie Zhu, Zhu Mao, Ayman H. Said, Bogdan M. Leu, Jinguang Cheng, Yoshiya Uwatoko, Changqing Jin, Jianshi Zhou

**Affiliations:** 1 Department of Geological Sciences, Jackson School of Geosciences, The University of Texas at Austin, TX 78712, USA; 2 Texas Materials Institute, The University of Texas at Austin, TX 78712, USA; 3 Center for High Pressure Science and Technology Advanced Research (HPSTAR), Shanghai, China; 4 Institute of Physics, Chinese Academy of Sciences, Beijing, China; 5 Laboratory of Seismology and Physics of Earth's Interior, School of Earth and Planetary Sciences, University of Science and Technology of China, Hefei, Anhui 230026, China; 6 Advanced Photon Source, Argonne National Laboratory, Argonne, IL 60439, USA; 7 Department of Mechanical Engineering, The University of Texas at Austin, TX 78712, USA; 8 Institute for Solid State Physics, The University of Tokyo, Kashiwa, Chiba 277-8581, Japan

## Abstract

Magnetite exhibits unique electronic, magnetic, and structural properties in extreme conditions that are of great research interest. Previous studies have suggested a number of transitional models, although the nature of magnetite at high pressure remains elusive. We have studied a highly stoichiometric magnetite using inelastic X-ray scattering, X-ray diffraction and emission, and Raman spectroscopies in diamond anvil cells up to ~20 GPa, while complementary electrical conductivity measurements were conducted in a cubic anvil cell up to 8.5 GPa. We have observed an elastic softening in the diagonal elastic constants (*C_11_* and *C_44_*) and a hardening in the off-diagonal constant (*C_12_*) at ~8 GPa where significant elastic anisotropies in longitudinal and transverse acoustic waves occur, especially along the [110] direction. An additional vibrational Raman band between the A_1g_ and T_2g_ modes was also detected at the transition pressure. These abnormal elastic and vibrational behaviors of magnetite are attributed to the occurrence of the octahedrally-coordinated Fe^2+^-Fe^3+^-Fe^2+^ ions charge-ordering along the [110] direction in the inverse spinel structure. We propose a new phase diagram of magnetite in which the temperature for the metal-insulator and distorted structural transitions decreases with increasing pressure while the charge-ordering transition occurs at ~8 GPa and room temperature.

Magnetite (Fe_3_O_4_) is the oldest known magnet that exhibits an inverse spinel structure with the chemical formula of Fe^3+^_(*Td*)_(Fe^2+^Fe^3+^)_(*Oh*)_O_4_ where subscripted *Td* represents the tetrahedral site occupied by Fe^3+^ ions and subscripted *Oh* indicates the octahedral site occupied equivalently by Fe^2+^ and Fe^3+^ ions^1^. Due to magnetite's natural geological occurrences in the Earth's crust and mantle as well as technological applications in magnetic and electronic materials and nano-composites, numerous experimental and theoretical studies have been conducted to understand its electronic, magnetic, and structural properties in extreme pressures and temperatures (*P-T*)^2^,^3^,^4^,^5^,^6^,^7^,^8^,^9^,^10^,^11^,^12^,^13^,^14^,^15^,^16^,^17^,^18^,^19^,^20^. Of particular interest is the occurrence of the metal-insulator Verwey transition at ambient pressure upon cooling below approximately 122 K, the Verwey temperature (*Tv*)^2^, which is strongly suppressed with increasing pressure up to approximately 8 GPa^7^,^8^,^9^,^14^,^15^. On the other hand, high-pressure studies at room temperature have also suggested a number of electronic, magnetic, and structural transitions in magnetite including the inverse-normal spinel transition^9^,^14^,^15^, the high-spin to intermediate-spin transition^16^,^19^, the enhanced delocalization or charge ordering of the 3*d* electrons of the iron ions^17^,^20^, the pressure-tuned ideal inverse-spinel structure^18^, as well as the occurrence of decreased bond length via changes in the local oxygen coordinates^13^. These proposed scenarios differ from each other significantly and entail very fundamental underlying physical mechanisms for understanding the behavior of magnetite at high pressures.

Based on the analyses of previous high-pressure Mössbauer spectra and refinements of the X-ray diffraction patterns^9^,^14^,^15^, it has been suggested that magnetite undergoes a transition from the inverse spinel structure to the normal spinel structure at pressures of approximately 10–20 GPa and room temperature, in which Fe^2+^ ions occupy the tetrahedral A site while all Fe^3+^ ions occupy the octahedral B site (Fe^2+^_(*Td*)_(Fe^3+^Fe^3+^)_(*Oh*)_O_4_). The inverse-normal spinel transition would result in a 50% increase in the net magnetic moment from 4 *μ_B_* to 6 *μ_B_* as well as significant local volume changes of the A and B-sites iron ions^9^,^14^,^15^. However, this normal spinel transition model was not confirmed by many other subsequent studies including Mössbauer and neutron studies using highly-stoichiometric magnetite samples^13^,^17^,^18^,^19^,^20^. On the other hand, combined high-pressure X-ray magnetic circular dichroism (XMCD) and X-ray emission spectroscopy (XES) studies on magnetite have suggested a reduction of the net magnetic moment of magnetite by approximately 50% at 12–16 GPa, indicating a high-spin to intermediate-spin transition of Fe^2+^ ions in the octahedral site^16^. However, such a spin-pairing model was soon disputed using results obtained from the same XMCD technique^19^.

As clearly pointed out in Verwey's original paper in 1939 and many later studies^1^,^21^,^22^,^23^, physical properties of magnetite in extreme *P-T* environments can be very sensitive to the stoichiometry, twinning, and presence of impurities and defects in the starting sample. Specifically, the metal-insulator transition near the *Tv* would occur continuously or discontinuously depending on the stoichiometry of the magnetite samples^1^,^21^,^22^; for a chemical formula Fe_3(1−δ)_O_4_ where δ is the degree of non-stoichiometry, the Verwey transition is of first order or second order when the absolute δ value is less than or more than 0.004, respectively. Twinning domains, on the other hand, have hampered the diffraction studies of the crystal structure^23^,^24^. Thus, care needs to be taken in preparing and characterizing the stoichiometry and avoiding twinning of the starting sample in order to better understand the behavior of magnetite at extreme environments. It should be noted that natural magnetite samples are intrinsically highly non-stoichiometric and can develop twinning domains^1^. Unfortunately, natural or synthetic magnetite samples have been commonly used in many of the literature studies without characterizing and reporting their non-stoichiometric δ values as well as the degree of twinning domains^1^, making a coherent comparison and understanding of the literature results extremely difficult.

A survey of previous studies at high pressures using highly stoichiometric starting samples shows that magnetite undergoes a transition at approximately 7–8 GPa and room temperature^13^,^18^,^20^. The abnormal changes in the hyperfine interaction parameters at 7 GPa have been interpreted as the occurrence of the decreased local oxygen internal coordinates (the decreased Fe-O bond length) at high pressures and room temperature^7^,^8^,^13^. However, high-pressure neutron diffraction results and *ab initio* calculations do not reveal any detectable change in the oxygen atomic parameter, yet show significant but continuous decrease of the magnetic moments at both the A and B sites to at least 10 GPa^17^. These results, nevertheless, rule out the possibility of a pressure-induced transition to the normal spinel structure at high pressures, and point to the possible occurrence of the charge-ordering of the iron ions at the B site^16^. Furthermore, a change in the curvature of the electrical conductivity at 6 GPa and room temperature has been attributed to be a result of the occurrence of the pressure-tuned ideal inverse-spinel structure^18^.

To decipher the behavior of magnetite at high pressures, here we have studied a highly stoichiometric and twinning-free single-crystal magnetite sample with a chemical formula Fe_3(1−δ)_O_4_, where |δ| = 0.00018 (Fig. S1), using high-energy resolution inelastic X-ray scattering (HERIX)^26^,^27^, X-ray diffraction (XRD), Raman spectroscopy, and X-ray emission spectroscopy (XES)^28^,^29^ measurements in diamond anvil cells (DACs) up to approximately 20 GPa (See Methods and SI for details)^22^,^25^,^30^. Complimentary electrical conductivity (EC) measurements were also conducted in a cubic anvil cell up to 8.5 GPa at room temperature (See SI for details). The HERIX and XRD results were used to derive full elastic constants of the single-crystal magnetite, while the Raman, XES, and EC results were used to understand the vibrational and electronic properties of the sample. These results show abnormal acoustic phonon and optical vibrational behaviors occurring at approximately 8 GPa. Using these experimental results and literature data, here we attribute the origin of the abnormal elastic and vibrational properties of magnetite at approximately 8 GPa to the local site distortions associated with charge-ordering in the inverse spinel lattice.

## Results

**Figure 1:**
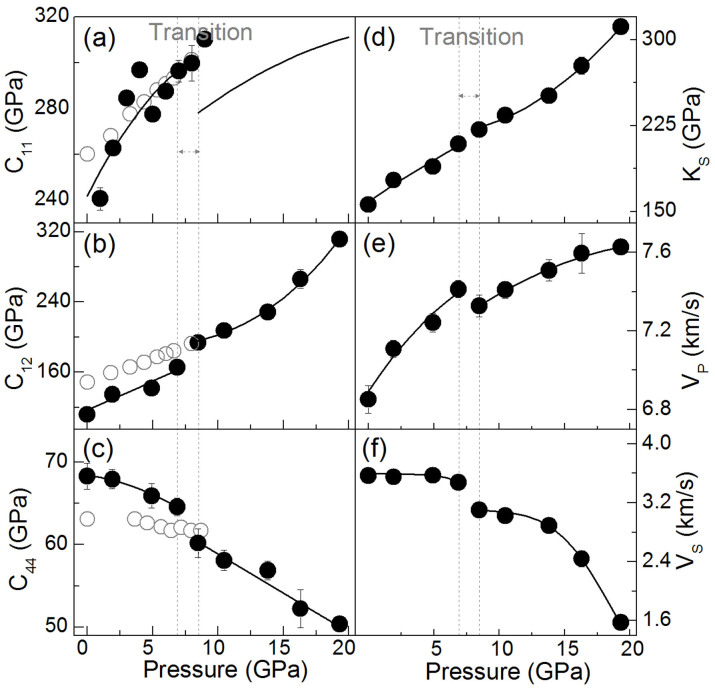
Elastic constants and sound velocities of single-crystal magnetite at high pressures. Solid circles: this study; open circles: Reichmann and Jacobsen (2004)^12^. Black lines represent simple polynomial fits to the experimental data, and are only meant to guide the eyes. Vertical lines indicate the transition pressure range at approximately 8 GPa. *Vp* and *Vs* represent the aggregate compressional and shear wave velocity, respectively, calculated from the single-crystal elasticity data.

High-pressure HERIX spectra of the single-crystal magnetite platelet with the pre-oriented (100) crystallographic plane were measured up to 19 GPa in a DAC for the lower momentum transfer (Q) part of the longitudinal acoustic (LA) and transverse acoustic (TA) phonon branches along [100] and [110] directions (See Methods and SI for details; Figs. S2–4)^26^,^32^. X-ray diffraction patterns were also collected to determine the crystallographic orientation matrix and to calculate the lattice parameter and the density of the sample as a function of pressure (Figs. S5; Table S1). The XRD patterns showed sharp reflections of the cubic inverse spinel structure without any observable structural transition, twinning domains, or volume collapse up to 20 GPa within the experimental uncertainties (Fig. S5). Using a linear fitting of the measured inelastic energy shift (E) as a function of Q near (800) and (440) reflection spots^26^, we have determined full elastic constants (*C_11_*, *C_12_*, and *C_44_*) along principle [100] and [110] axes for the longitudinal (*Vp*) and transverse (*Vs*) acoustic waves based on the following equations: 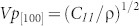

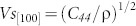

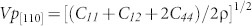

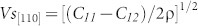
where ρ is the density and the subscript [uvw] indicates the crystallographic direction of the acoustic phonon wave propagation (Fig. 1)^32^. Using these elastic constants, we have also derived adiabatic bulk modulus (*K_S_*), shear modulus (*G*), and aggregate *Vp* and *Vs* of the single crystal^30^.

The *C_11_* and *C_12_* elastic constants show monotonic increases with increasing pressure up to approximately 7 GPa, while the *C_44_* decreases with increasing pressure (Fig. 1). Between 7 and 8.5 GPa, abnormal changes in the constants and sound velocities occur in which the diagonal elastic constants *C_11_* and *C_44_* drop by approximately 6–7%, respectively, while the off-diagonal elastic constant *C_12_* jumps by 17% (Fig. 1). The aggregate *Vp*, *Vs* and *G* also decrease significantly within this pressure interval. However, the *K_S_* continuously increases with increasing pressure normally up to 19 GPa and does not show any visible change at the transition pressure range for other elastic constants; this behavior of the *K_S_* as a function of pressure is consistent with the compressional pressure-volume curve derived from the X-ray diffraction spectra as a function of pressure (Fig. S5). Between 16 GPa and 20 GPa, the *C_12_*, *G*, and *Vs* also significantly decrease with increasing pressure. The softening of these constants between 16 GPa and 20 GPa in the inverse spinel phase can be attributed to its approaching the previously reported orthorhombic phase at approximately 22 GPa^3^,^5^,^6^.

**Figure 2:**
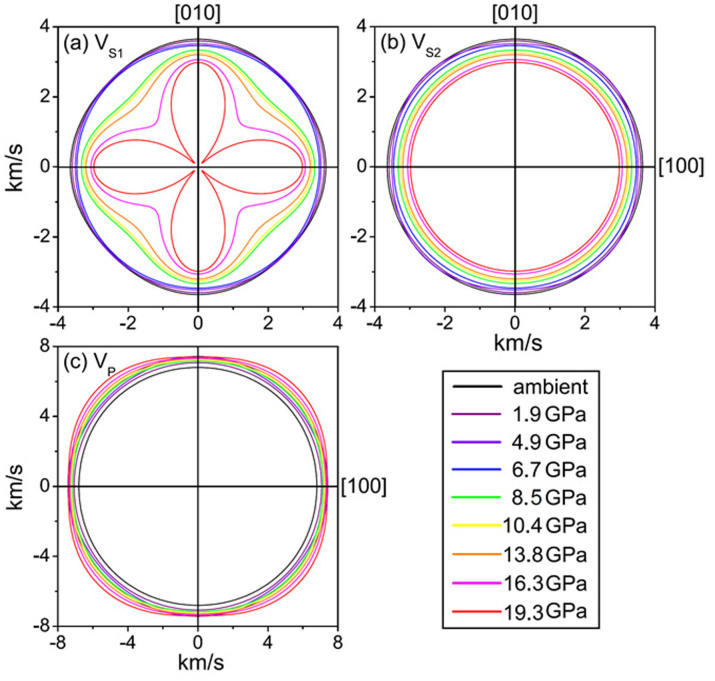
Compressional and shear wave anisotropies of single-crystal magnetite at high pressures. The notation for the two polarized shear waves is defined as *V_S1_* being slower in velocity than *V_S2_*. Principle axis [100] is along the horizontal line while the vertical direction is [010]. *Vp* and *V_S_* velocities exhibit an enhanced anisotropy starting at 8.5 GPa. Specifically, the *V_S1_* and *Vp* change dramatically along the [110] direction across the transition at approximately 8 GPa.

To understand the abnormal behavior of the elasticity as a function of pressure in detail, we have also calculated the *Vp*, *Vs*_1_, and *Vs*_2_ as a function of the crystallographic direction at high pressures (Figs. 2, S4)^32^. The *Vp* and *Vs*_1_ exhibit significant changes especially along the [110] direction between 7 and 8.5 GPa with a significantly increased anisotropy factor (*A*) (Fig. 2). The *V_S_* splitting anisotropy factor (*A_Vs_*) jumps from a few percent to 17% while the *V_p_* anisotropy factor (*A_Vp_*) increases from approximately 1% to 4% (Fig. S4), indicating that the single-crystal magnetite becomes highly elastically anisotropic at pressures above approximately 8 GPa. We note that X-ray diffraction patterns of the sample in the soft Ne medium showed sharp and round diffraction spots with well converged lattice parameters, ruling out the possibility that the observed anisotropy was induced by the non-hydrostaticity in the sample chamber.

**Figure 3:**
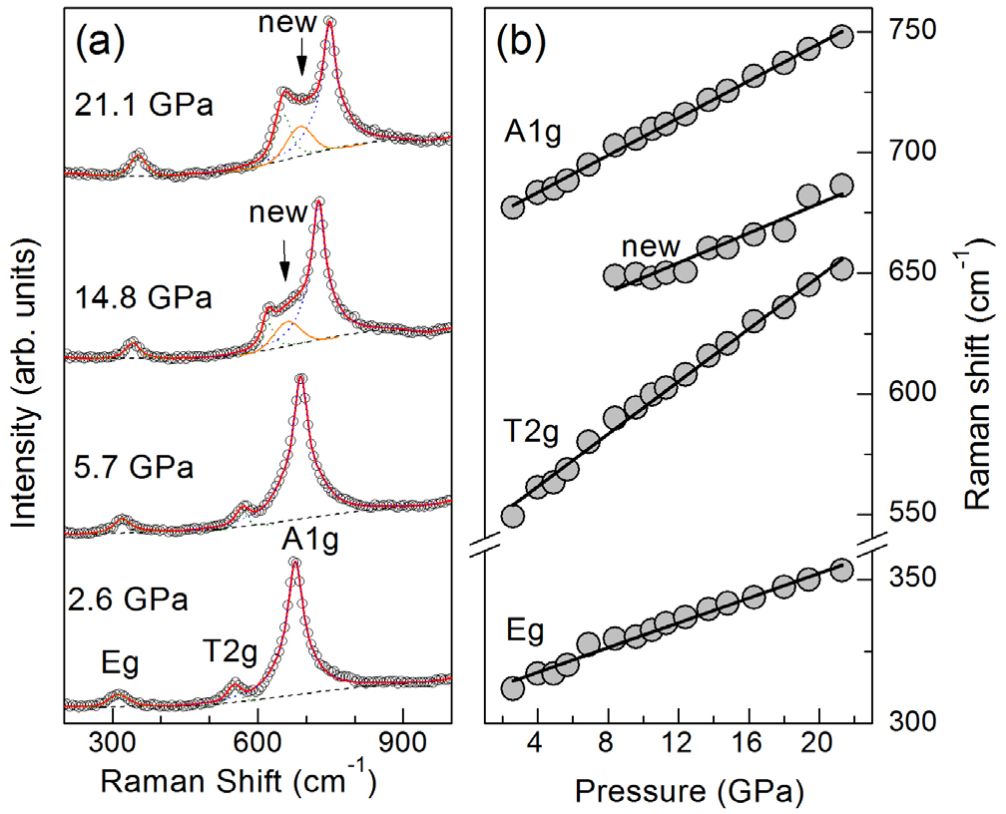
Vibrational Raman modes of single-crystal Fe_3_O_4_ at high pressures. (a) Representative Raman spectra; (b) derived Raman frequencies. A new Raman band occurs between *A_1g_* and *T_2g_* modes (orange line) starting at approximately 8 GPa.

To decipher the observed abnormal transition in the elasticity of magnetite, we have also conducted complimentary Raman spectroscopy, synchrotron Fe *K_β_* XES, and the bulk electrical conductivity (EC) experiments at high pressures (See Methods and SI, Fig. 3, S6–7)^28^,^29^. Analyses of the high-pressure Raman spectra show three distinct Raman bands that can be assigned to *A_1g_*, *T_2g_*, and *E_g_* modes of the cubic inverse spinel structure up to 21 GPa (Fig. 3)^10^,^11^,^33^,^34^. Raman frequencies of these modes increase almost linearly with increasing pressure up to the highest pressure of 21 GPa, although the relative intensity of the *T_2g_* mode with respect to the *A_1g_* mode starts to increase with increasing pressure at approximately 8 GPa (Fig. 3a). Most significantly, an additional Raman band occurs in the spectral range between the *A_1g_* and *T_2g_* bands, and its Raman frequency also increases linearly with increasing pressure, similar to that of other magnetite Raman bands (Fig. 3).

Analyses of the synchrotron Fe *K_β_* X-ray emission spectra using the absolute and relative integrated areas methods proposed previously^28^,^29^ show that the total spin momentum (*S*) of all iron ions slightly decreases with increasing pressure without any observable discontinuous changes up to 19 GPa (Fig. S6). Based on these spectral analyses, the slight continuous decrease in the intensity of the satellite *K_β_*′ can be attributed to the spectral broadening effect, instead of any spin-pairing transition of iron in magnetite (Fig. S6)^16^,^19^. Electrical resistance of the single-crystal magnetite as a function of pressure measured in a cubic anvil cell continuously decreases with increasing pressure (Fig. S7), and a continuous change in the curvature can be identified at approximately 7 GPa, consistent with previous results^7^,^8^,^18^. The electrical resistance results indicate that magnetite becomes a better metallic conductor at higher pressures.

## Discussion

Previous high-pressure ultrasonic interferometry and Raman measurements^10^,^11^,^12^ on highly non-stoichiometric natural magnetite samples did not reveal any changes in elasticity nor the occurrence of the additional Raman band at the pressure range of 7–8 GPa where we observed the occurrence of the abnormal elastic and vibrational behaviors on a highly stoichiometric magnetite sample. Our elasticity results also differ from the high-pressure ultrasonic interferometry results^12^ on a natural single-crystal magnetite by approximately 10%, although the differences become smaller at higher pressures (Fig. 1). Thus, it is evident that the differences in these observations can be attributed to the stoichiometry of the samples used in these studies; while our sample was highly stoichiometric with a chemical composition of Fe_3(1−δ)_O_4_ where |δ| = 0.00018,^25^ the natural magnetite sample used in the ultrasonic study was likely non-stoichiometric^12^. Together with the inconsistency in the literature results on other physical properties of magnetite at high pressures, it is clear that the high-pressure behavior of magnetite is highly sensitive to the stoichiometry of the starting sample and that the abnormal transition at approximately 8 GPa only occurs in highly-stoichiometric samples. Therefore, experimental results obtained from highly stoichiometric samples are of direct relevance to understanding the high-pressure behavior of the sample.

Based on our X-ray emission results, we can clearly rule out the spin-pairing transition model of the iron ions in magnetite at high pressures^16^,^19^. The inverse to normal spinel transition model^9^,^14^,^15^ is also unlikely to contribute to our observed abnormal elastic and vibrational behavior, because such a transition would result in significant changes in the local site symmetry and volumes of the lattice as well as in the electrical transport property; all of which were also not observed here nor in previous studies using highly stoichiometric samples^7^,^8^,^13^,^17^,^18^,^20^.

Based on the group theory and lattice-vibration analyses, the inverse spinel structure of magnetite in the primitive unit cell contains Fe and O atoms in three distinct site symmetries (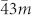 (*T_d_*), 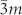 (*D_3d_*) and 3m (*C_3V_*)), in which the *T_d_* and *C_3V_* sites occupied by Fe^3+^ and O^2+^ ions, respectively, contribute to the five Raman-active modes of the FeO_4_ tetrahedron including one *A_1g_*, one *E_g_*, and three *T_2g_* (Fig. 3)^10^,^11^,^33^,^34^. The *A*_1*g*_ mode at ~668 cm^−1^ arises from the symmetric stretch of the O atoms along Fe-O bonds, the *T*_2*g*_ mode at ~538 cm^−1^ is the asymmetric stretching of the Fe and O atoms, and the *Eg* mode at ~306 cm^−1^ comes from the symmetric bonds between Fe and O atoms. Due to the delocalized nature of the indistinguishable Fe^2+^ and Fe^3+^ 3*d* electrons in magnetite at ambient conditions, the octahedral site retains the local 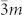 (*D_3d_*) symmetry, which would otherwise exhibit a lower symmetry due to the occupation of both Fe^2+^ and Fe^3+^ in the more “ideal” inverse spinel structure^33^,^34^. Such charge transfer also contributes to the “half metal” electrical conduction behavior of magnetite through the overlapping integrals between Fe^2+^ and Fe^3+^ ions via the octahedral network above the Verwey transition temperature at ambient pressure^7^,^8^,^18^.

**Figure 4:**
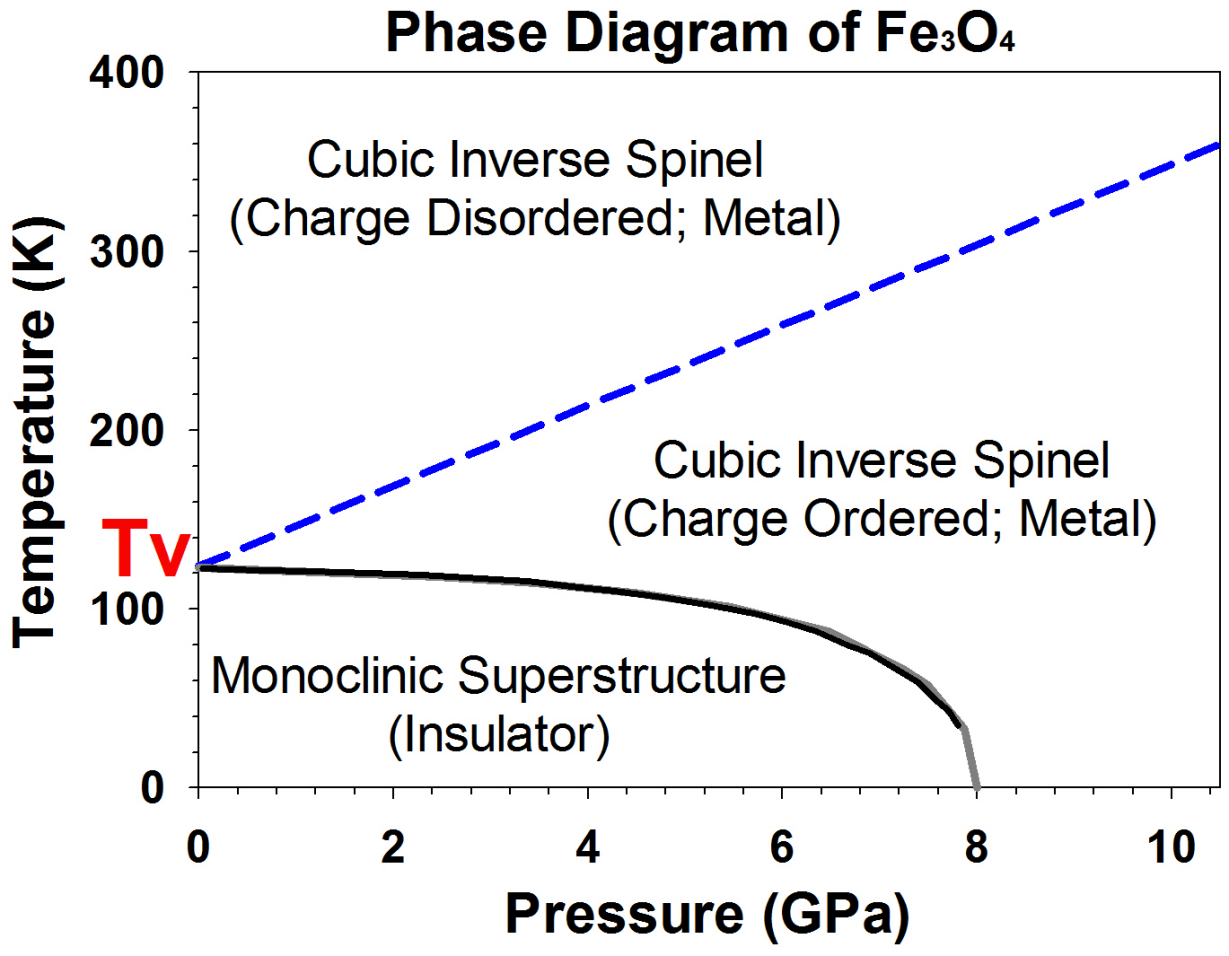
Phase diagram of Fe_3_O_4_ at extreme pressure and temperature. The diagram is constructed using our results as well as literature results^2^,^4^,^7^,^8^,^9^,^13^,^14^,^15^,^18^,^20^,^21^. Magnetite undergoes the Verwey transition (*Tv*) at ambient pressure where structural, charge ordering, and metal-insulator transitions occur. The temperature for the metal-insulator transition (solid grey line)^7^,^8^ and the distorted structural transition (solid black line)^14^ decreases with increasing pressure, while the temperature for the charge ordering transition (blue dashed line) increases with increasing pressure^9^,^13^,^18^,^20^.

Recently, it has been reported from detailed X-ray diffraction structural analyses of a single-crystal magnetite that Fe^3+^-Fe^2+^-Fe^3+^ ions at the B-site form linear three-Fe-site units, called trimerons, with the charge order and three-site distortions along the [110] direction below *Tv* (Ref. 23). The formation of the trimerons can induce substantial atomic displacements and affect the coupling between the large electrical polarization and the magnetization^23^. It has also been proposed that these trimerons may become important quasiparticles in magnetite above the Verwey temperature^23^. Examinations of the measured *Vp* and *V_S_* velocities as well as the derived *Cij* of the single-crystal magnetite indeed show much more dramatic changes in the *V_S1_* and *Vp* velocities along the [110] direction across the transition at approximately 8 GPa than other directions (Figs. 1, 2). The pressure-induced isostructural transition at 8 GPa with enhanced anisotropic elasticity suggests that the Fe^3+^-Fe^2+^ charges at the B-site become ordered at pressures above 8 GPa. It should be noted here, however, that the Verway transition is accompanied with the trimeron ordering, the monoclinic structural transition, and the metal-insulator transition at ambient pressure^1^ (Fig. 4). As the transition to the trimeron ordered phase is separated from the metal-insulator transition at increasing pressures (Fig. 4), there is no longer a dramatic change in the electrical transport property. Ordered trimerons would result in a less symmetrical yet “ideal” inverse spinel structure with a small displacement (distortion) along the [110] direction which would contribute to additional Raman modes and would affect the elastic constants and sound velocities along the direction more significantly than other directions^33^. Thus, the iso-symmetric electronic transition model from the charge delocalized state to the ordered trimeron in the octahedral site of magnetite at approximately 8 GPa is most consistent with our observations and literature results from highly stoichiometric samples. Our observed transitional pressure is also consistent with the Mössbauer studies on highly stoichiometric magnetite samples, which showed significant changes in the hyperfine parameters, especially in the octahedral sites^9^,^13^,^14^,^15^.

The strength of the electron-phonon interaction in magnetite plays an important role in its unique electronic and magnetic properties at ambient conditions. It is conceivable that the occurrence of the ordered electronic transition at approximately 8 GPa significantly affects the electron-phonon interaction, resulting in the abnormal acoustic phonon behavior in the inverse spinel structure observed in the HERIX measurements. The softened *C_11_* (diagonal compressional component) and *C_44_* (diagonal shear component) and enhanced *C_12_* (off-diagonal component) observed here would thus hold the key to deciphering this localized electronic transition. Since previous studies have suggested that the transition in magnetite at approximately 8 GPa and 300 K can extend very close to the Verwey transition at approximately 122 K and ambient pressure^7^,^8^,^9^,^14^,^15^ (Fig. 4), future investigations at high pressure and low-temperature conditions are needed to decipher the relationship between the Verwey transition and the transition to the trimeron ordered phase in the inverse spinel structure.

## Methods

Synthesized single-crystal magnetite with a highly stoichiometric chemical composition of Fe_3(1−δ)_O_4_, where |δ| = 0.00018, was used for all of our high-pressure experiments (SI; Figs. S1–7)^22^,^25^. A millimeter-size piece of the single-crystal magnetite was oriented in the (100) plane using single-crystal X-ray diffraction (XRD) at the Texas Materials Institute of the University of Texas at Austin. The oriented sample of approximately 150 μm in diameter was then double-side polished to 40 μm in thickness. A Re gasket of 5 mm in diameter and 250 μm in thickness was pre-indented to 15 GPa using a pair of diamond anvils having 500 μm flat culets in a short symmetric DAC. A hole of 250 μm was drilled on the pre-indented area of approximately 75 um thick, and then used as the sample chamber. The sample was loaded into the sample chamber, together with two ruby spheres as the pressure calibrant^29^. Ne gas was loaded into the sample chamber as the pressure medium for inelastic and X-ray diffraction experiments using the Gas Loading System at the Mineral Physics Laboratory of the University of Texas at Austin.

High-energy resolution inelastic X-ray scattering (HERIX)^26^,^27^ and XRD experiments were conducted in a short symmetric diamond anvil cell (DAC) up to 19 GPa at 300 K at Sector 30 of the Advanced Photon Source (APS), Argonne National Laboratory. The DAC has an opening of approximately ±40° for the experiments in the axial direction. An incident X-ray beam with an energy of approximately 23.724 keV and an energy bandwidth of 1.5 meV was focused to a beamsize of 15 × 35 μm at the sample^27^. An online image plate diffraction detector was used to orient the crystal with the (800) or (440) diffraction spot aligned in the horizontal plane. The scattered inelastic signals of the sample were collected and analyzed using a spherically-bent silicon crystal analyzer of the (12 12 12) reflection and a detector working very close to back reflection (89.98°) in the energy range of ±10–12 meV with a step size of 0.5 meV^31^. Typically, the HERIX spectra for the longitudinal and transverse acoustic phonons were collected along the [100] and [110] directions with a spectral energy resolution of approximately 1.5 meV and a momentum resolution of 0.2 nm^−1^ (Figs. S2, S3). The collection time for each energy scan was approximately 2 hours and about 3–4 spectra were co-added for each set of experimental conditions. XRD diffraction spots of the sample were also collected and used to determine the sample densities as a function of pressure, while an online ruby system was used to measure the ruby fluorescence for pressure determinations (Fig. S5).

High-pressure Raman spectroscopic measurements on a (100)-oriented single-crystal magnetite were conducted in a symmetric DAC having a pair of 200 μm diamond anvils which consisted of a natural ultralow-fluorescence diamond anvil and a synthetic ultralow-fluorescence diamond anvil produced by Sumitomo Corporation. Since the Raman signals of the opaque magnetite were extremely weak, the use of the synthetic diamond with extremely low fluorescence background significantly enhanced the signal-to-noise ratio of the Raman spectra. Similar to the HERIX experiments, the sample was loaded into the sample chamber of a DAC with two ruby spheres as the pressure calibrant^30^ and Ne gas as the pressure medium. A Renishaw inVia Raman Microscope system equipped with a 532 nm laser and a long-working distance lens of 20× was used for collecting the Raman spectra with a typical data collection time of 30 mins. The HERIX and Raman spectra were analyzed using the OriginPro 8 Program.

## Author Contributions

J.F.L. conceived & designed the research; J.F.L., J.W., J.Z., A.S., B.M.L., J.C., Y.U. and J.Z. conducted the high-pressure experiments; Z.M. and J.W. analyzed the X-ray data; J.Z. analyzed the Raman data; J.F.L., C.J. and J.Z. wrote the paper.

## Supplementary Material

Supplementary Information Supplementary Information
