# Supplementary material for: Abnormal Elastic and Vibrational Behaviors of Magnetite at High Pressures
Source: Sci Rep. 2014 Sep 4;4:6282. doi: 10.1038/srep06282 (PMC4153994; doi:10.1038/srep06282)
Supplement: Supplementary Information [file srep06282-s1.pdf]

# Abnormal Elastic and Vibrational Behaviors of Magnetite at High Pressures

Jung-Fu Lin[a,b,c], Junjie Wu[c,d], Jie Zhu[d], Zhu Mao[a,e], Ayman H. Said[f], Bogdan M. Leu[f], Jinguang Cheng[b,g,h], Yoshiya Uwatoko[h], Changqing Jin[d], Jianshi Zhou[b,g]

[a]*Department of Geological Sciences, Jackson School of Geosciences, The University of Texas at Austin, TX 78712, USA*

[b]*Texas Materials Institute, The University of Texas at Austin, TX 78712, USA*

[c]Center for *High Pressure Science and Technology Advanced Research (HPSTAR), Shanghai, China*

[d]*Institute of Physics, Chinese Academy of Sciences, Beijing, China*

[e]*Laboratory of Seismology and Physics of Earth's Interior, School of Earth and Planetary Sciences, University of Science and Technology of China, Hefei, Anhui 230026, China*

[f]*Advanced Photon Source, Argonne National Laboratory, Argonne, IL 60439, USA*

[g]*Department of Mechanical Engineering, The University of Texas at Austin, TX 78712, USA*

[h]*Institute for Solid State Physics, The University of Tokyo, Kashiwa, Chiba 277-8581, Japan*

*Correspondence and requests for materials should be addressed to J.F.L (afu@jsg.utexas.edu)*

## Supporting Information

SI Text

**Single-crystal synthesis and characterization:** Single-crystal growth of the starting magnetite was carried out in an image furnace (NEC model SC-M35HD) in the atmosphere of high-purity argon (99.999% purity) at the Department of Mechanical Engineering, the University of Texas at Austin. The feed and seed rods, made of $Fe_3O_4$ powder (99.999% purity), were first loaded into a rubber tube and were compressed to 4 kbar of hydrostatic pressure. The compacted rods were then sintered in the furnace at 937 ºC with an $O_2$ gas flow for 20 hrs. During an estimated crystal growth rate of 8 mm/h, the feed and seed rods were rotated in opposite directions with a rotation

speed of 30 rpm each. Laue X-ray back diffraction patterns were used to measure lattice parameters and orientations of the synthesized single crystal, which confirmed the cubic space group of $Fd\bar{3}m$ with the lattice parameter $a$ = 8.3964 Å. Temperature-dependent measurements on the specific heat and resistivity of the synthesized single-crystal magnetite showed that the Verwey transition occurred sharply with the first-order characteristics at 122 K (Fig. S1)[1,2]. Based on these analyses, the synthesized single crystal has a chemical composition of $Fe_{3(1-\delta)}O_4$ with $|\delta| = 0.00018$, which is a nearly ideal chemical stoichiometry (Fig. S1)[1,2].

**X-ray emission spectroscopy (XES) experiments:** A Be gasket of 3 mm in diameter was pre-indented to 20 GPa using a pair of diamond anvils having 400 μm culets in a symmetric DAC. A hole of 150 μm was drilled in the very center of the pre-indented area and subsequently used as the sample chamber. A single-crystal magnetite approximately 50 μm in diameter was polished down to a 15 μm disk in thickness, and then loaded into the sample chamber, together with mineral oil as the pressure medium and a few ruby spheres of approximately 10 μm in diameter as the pressure calibrant[3]. High-pressure XES measurements were performed at the HPCAT Sector of the APS, ANL. An incident X-ray beam with an incident energy of 11.3 keV and a bandwidth of approximately 1 eV was used for the experiments[4]. The collection time for each XES spectrum was approximately 30 minutes, and 4 to 6 spectra were added together for a given pressure. $FeS_2$ was used as the low-spin reference, while the sample at ambient conditions and hematite were used as the high-spin reference[4-6] (Fig. S4). Analyses of the XES were performed using both Integrated Absolute Difference (IAD) and Integrated Relative Difference (IRD) methods[4-6]. The derived total spin momentum shows a continuous decrease with increasing pressure up to approximately 20 GPa, but such a decrease can be attributed to the spectral broadening effect[4], instead of any electronic spin-pairing transition[7].

**References:**


1. Shepherd JP, Koenitzer JW, Aragon R, Spalek J, Honig JM (1991) Heat capacity and entropy of nonstoichiometric magnetite $Fe_{3(1-\delta)}O_4$: The thermodynamic nature of the Verwey transition. *Phys Rev B* 43: 8461-8471.

2. Zhou J-S, Goodenough JB, Dabrowski B (2005) Pressure-induced non-Fermi-liquid behavior of $PrNiO_3$. *Phys Rev Lett* 94: 226602.

3. Mao HK, Xu J, Bell PM (1986) Calibration of the ruby pressure gauge to 800-Kbar under quasi-hydrostatic conditions. *J Geophys Res* 91: 4673-4676.

4. Mao Z, et al. (2014) Spin and valence state of iron in Al-bearing silicate glass at high pressures studied by synchrotron Mössbauer and X-ray emission spectroscopy. *Am Miner* 99: 415-423.

5. Lin JF, Speziale S, Mao Z, Marquardt H (2013) Effects of the electronic spin transitions of iron in lower-mantle minerals: implications to deep-mantle geophysics and geochemistry. *Rev Geophys* 51: 244-275.

6. Vanko G, et al. (2006) Probing the 3*d* spin momentum with X-ray emission spectroscopy: The case of molecular-spin transitions. *J Phys Chem B* 110: 11647-11653.

7. Ding Y, et al. (2008) Novel pressure-induced magnetic transition in magnetite ($Fe_3O_4$). *Phys Rev Lett* 100: 045508.

8. Birch F (1947) Finite elastic strain of cubic crystals. *Phys Rev* 71: 809-824.

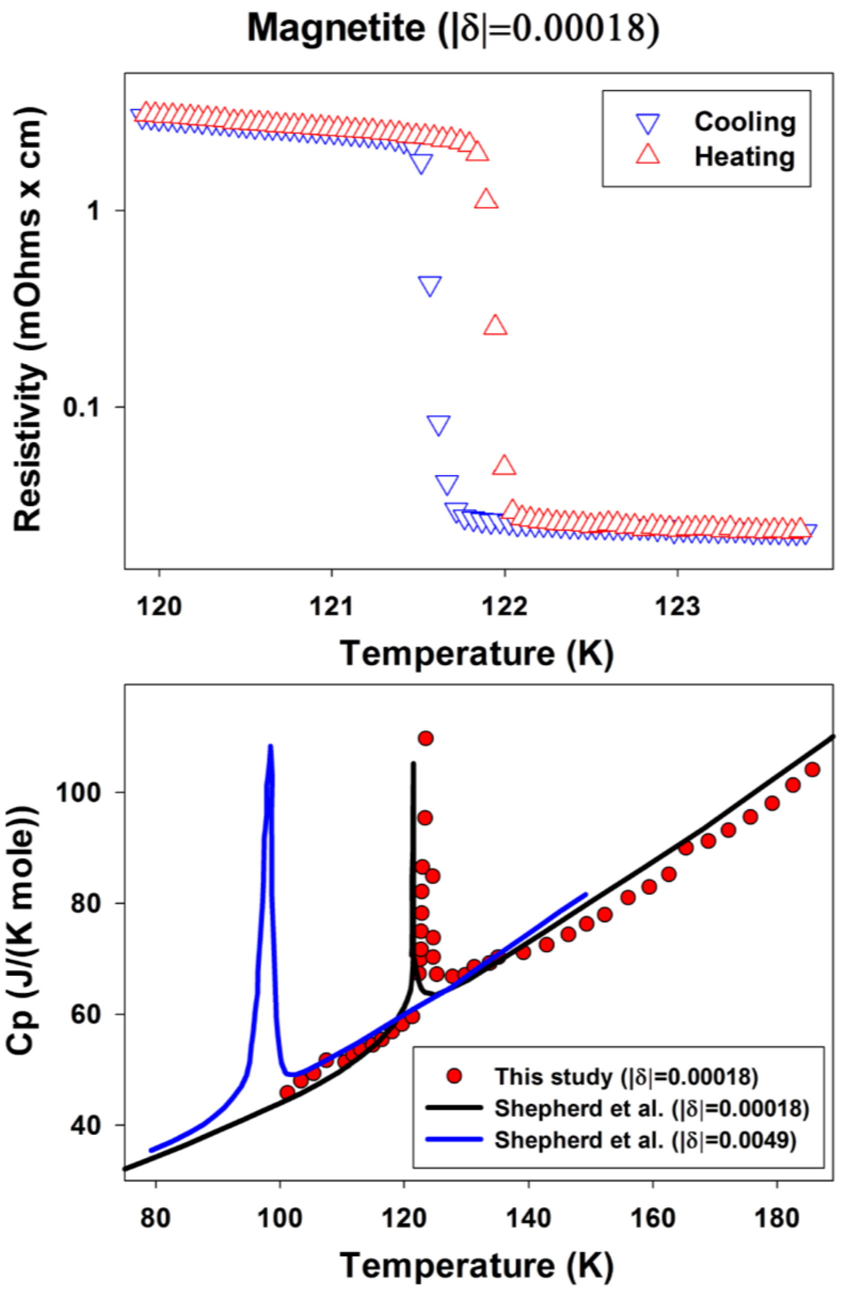


**Figure S1.** Resistivity and heat capacity of single-crystal magnetite as a function of temperature. Compared with the literature results[1], the measurements show that the sample was very close to stoichiometric with a chemical formula $Fe_{3(1-\delta)}O_4$ where $|\delta| = 0.00018$. Literature results by Shepherd et al.[1] were used for the derivation of the δ value[2].

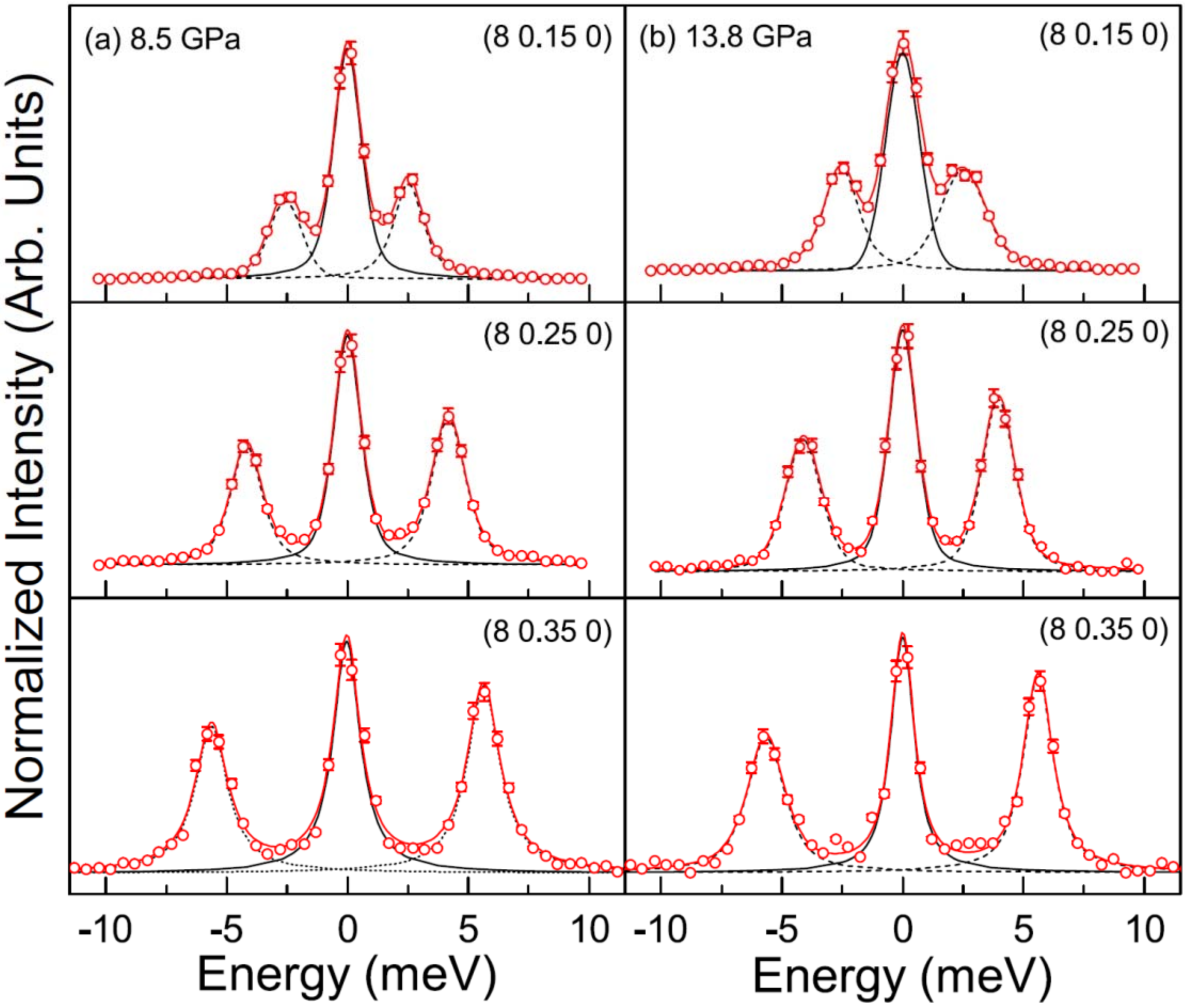


**Figure S2.** Representative high-energy resolution inelastic X-ray scattering spectra of single-crystal magnetite at high pressures. (**a**) 8.5 GPa; (**b**) 13.8 GPa. Longitudinal acoustic phonons (red circles) were measured at three momentum transfers of (8 0.15 0), (8 0.25 0), and (8 0.35 0) in the energy range of $\pm$ 10 meV with a step size of 0.5 meV. Solid black line: modeled elastic peak; dashed black lines: modeled longitudinal acoustic peak for phonon addition and annihilation, respectively.

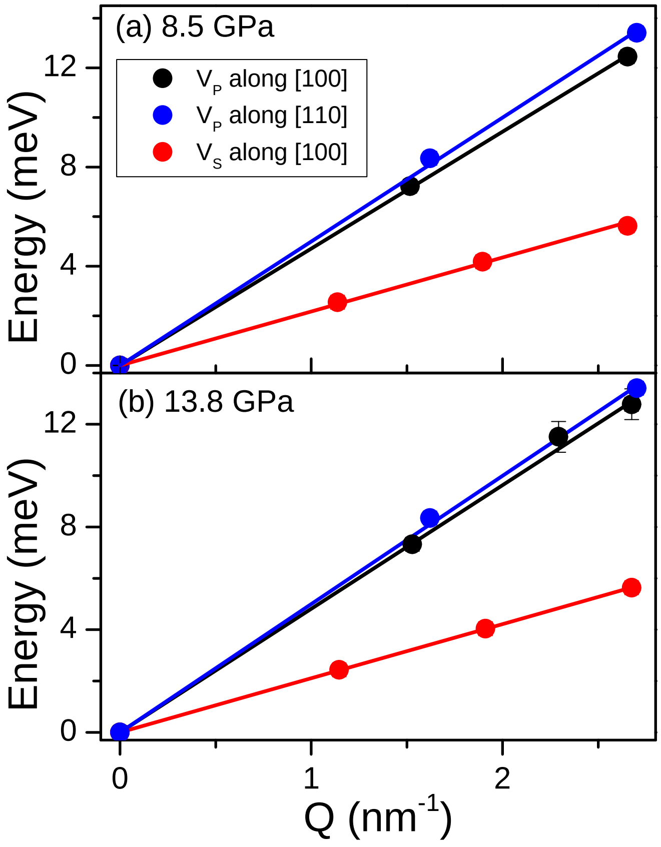


**Figure S3.** Representative acoustic phonon energy of single-crystal magnetite as a function of the momentum transfer along the [100] and [110] directions. Some error bars are smaller than the solid circles when not shown.

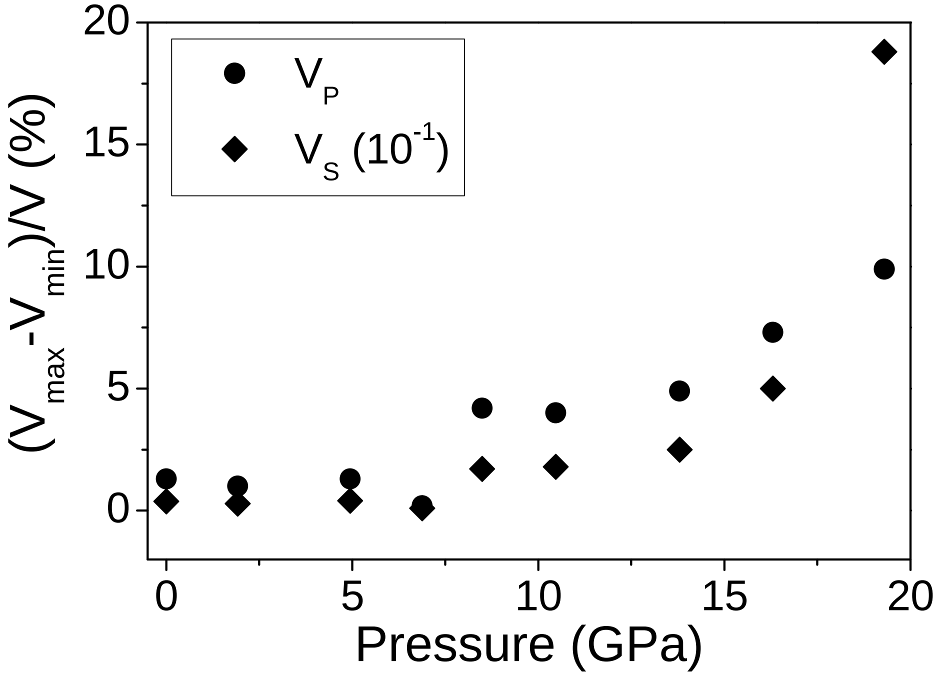


**Figure S4.** Aggregate *Vp* and *Vs* anisotropy of magnetite as a function of pressure. The anisotropy factor in percentage for *Vp* is derived from the difference between the maximum velocity ($V_{max}$) and the minimum velocity ($V_{min}$), while the *Vs* anisotropy is the shear wave splitting anisotropy between $V_{S1}$ and $V_{S2}$.

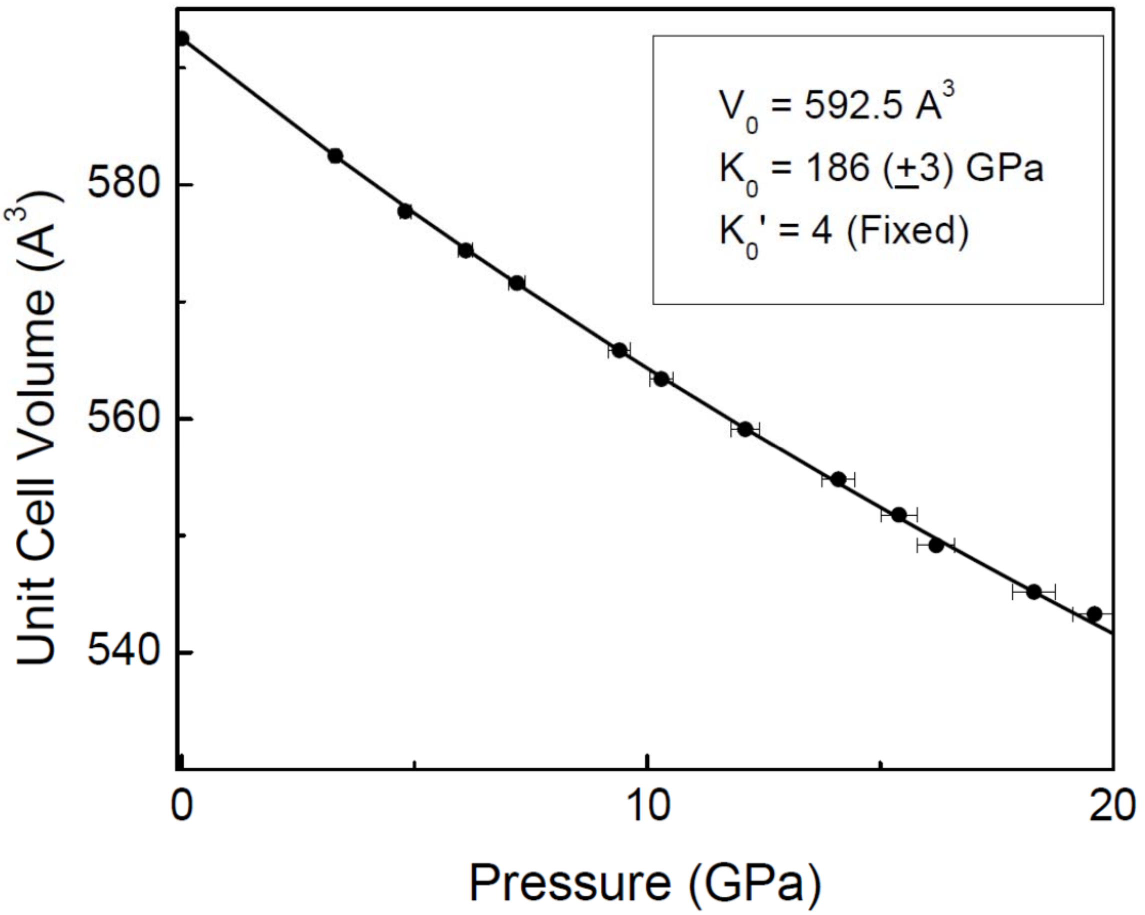


**Figure S5.** Pressure-volume relation of single-crystal magnetite at high pressures. The results up to 20 GPa can be well modeled by the Birch-Murnaghan equation of state with the pressure derivative of the bulk modulus ($K_0'$) fixed at four[8]. Error bars are not shown when smaller than the solid circles.

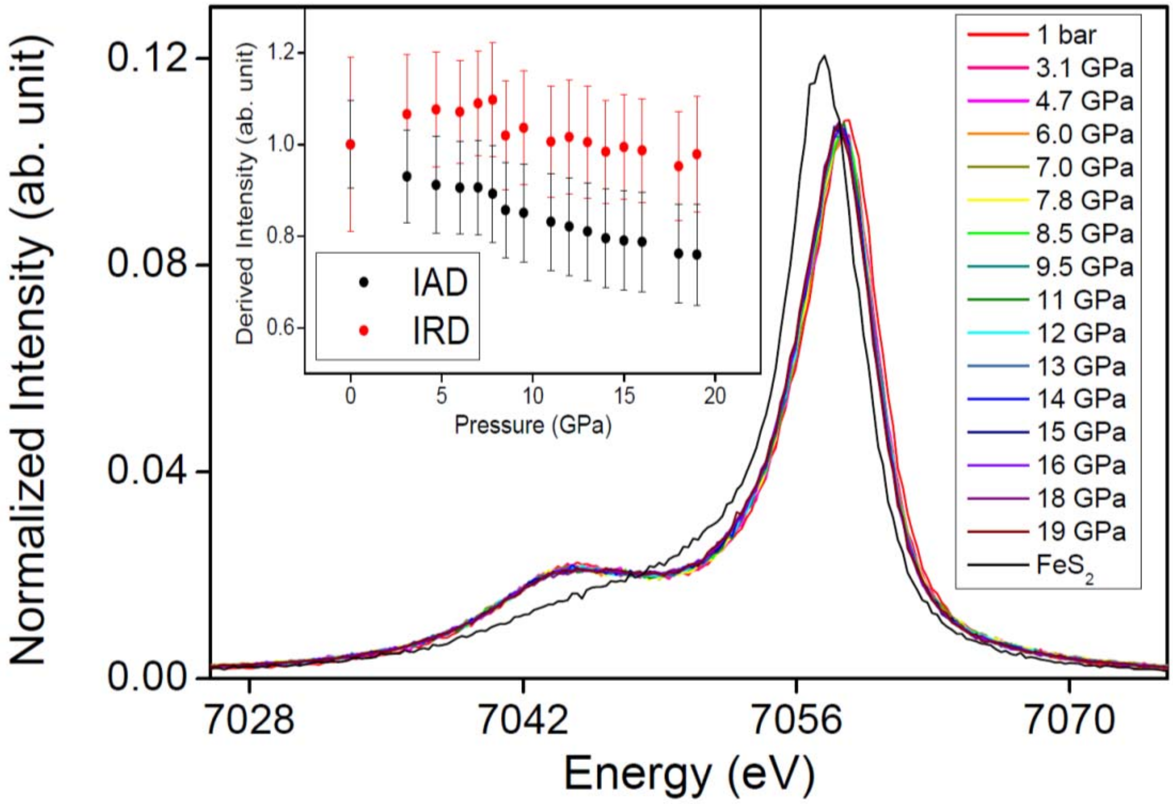


**Figure S6.** Fe $K_\beta$ X-ray emission spectra of single-crystal magnetite at high pressures. $FeS_2$ is used as the spectral reference for the low-spin state, while the sample itself was used as the high-spin reference[4-6.]

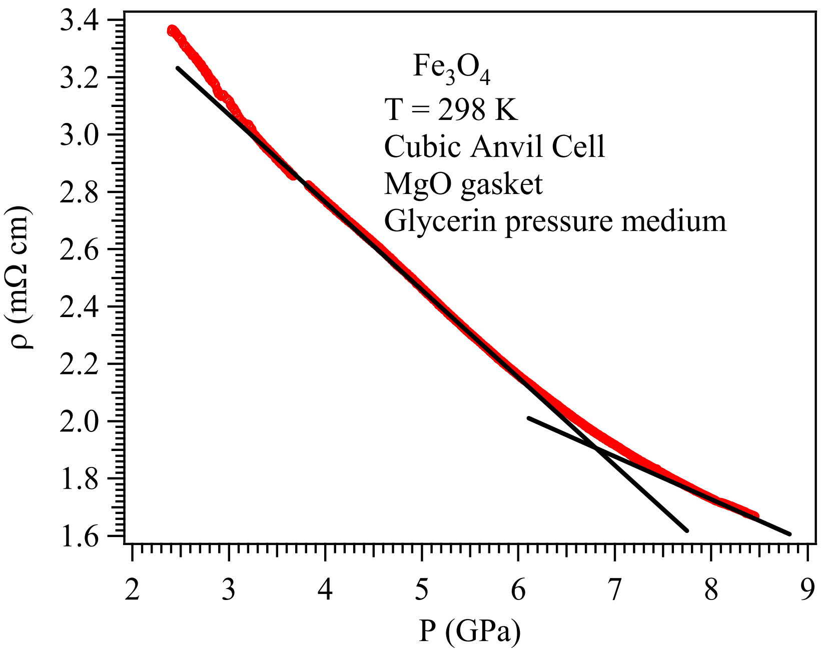


**Figure S7.** Electrical resistance of single-crystal magnetite as a function of pressure. The measurements were conducted in a cubic anvil cell having MgO gasket and glycerin pressure medium at room temperature.

**Table S1.** Elastic constants of single-crystal magnetite at high pressures.

| P (GPa) | $C_{11}$ (GPa) | $C_{44}$ (GPa) | $C_{12}$ (GPa) | $K_S$ (GPa) | G (GPa) |
|---|---|---|---|---|---|
| 0.0 | 240.6 (±4.9) | 68.3 (±1.6) | 113.8 (±5.9) | 156.0 (±4.3) | 66.3 (±1.8) |
| 1.9 | 262.8 (±3.0) | 67.9 (±1.2) | 134.6 (±3.9) | 177.4 (±2.8) | 66.4 (±1.2) |
| 4.9 | 284.5 (±2.7) | 65.9 (±1.5) | 141.7 (±4.7) | 189.3 (±3.3) | 68.1 (±1.4) |
| 6.9 | 296.8 (±2.8) | 64.6 (±1.1) | 165.3 (±4.5) | 209.1 (±3.1) | 65.0 (±1.2) |
| 8.5 | 277.5 (±2.7) | 60.2 (±1.8) | 193.6 (±5.6) | 221.6 (±3.8) | 52.1 (±1.6) |
| 10.5 | 287.4 (±3.1) | 58.1 (±1.3) | 207.2 (±4.3) | 234.0 (±3.1) | 50.1 (±1.3) |
| 13.8 | 296.3 (±4.8) | 56.9 (±1.1) | 228.4 (±5.5) | 251.0 (±4.0) | 46.2 (±1.6) |
| 16.3 | 299.8 (±7.7) | 52.2 (±2.0) | 266.1 (±10.7) | 277.3 (±7.6) | 33.2 (±3.0) |
| 19.3 | 310.2 (±2.1) | 50.4 (±0.9) | 311.6 (±3.9) | 311.2 (±2.7) | 14.0 (±1.0) |
